# Prof. Parker’s Clinique, College of Physicians and Surgeons, New York

**Published:** 1847-08

**Authors:** 


*Prof. Parker's Clinique, College of Physicians and Surgeons, New-York.*

*Case* 1*st.*—*Rheumatism.*—A boy of 15, after being exposed to cold and wet, was seized with pain in the back, shoulders, thighs, and knees. Complains most of right knee, which is swollen from slight effusion into the joint, causing a limp in the walk. The tongue is coated, bowels not free, urine somewhat scanty and rather higher color than natural. *Remarks by Dr. P.*—The pain in both thighs and knees might induce the suspicion of some spinal disease, but examination elicits nothing further to warrant it. The three affections which are apt to present themselves in the knee are acute synovitis, scrofulous synovitis and rheumatism. Having arrived at the conclusion, in my own mind, that this boy has the latter, let me give you my reasons for this opinion. Rheumatism, like gout, is a constitutional disease, having its origin in morbid function of the digestive apparatus. In its articular visitations, it rarely confines itself to a single joint, but attacks several simultaneously or in succession. The secretions generally are changed, the perspiration and urine becoming in some cases strongly acid, and the latter containing lithic acid deposites. The violence of the general symptoms depends, in some degree, upon the nature of the tissues involved, being more intense where the disease attacks the *fibrous* membranes near the joint, than in the *synovial* variety. A distinction in the local appearance is also observed in these two forms of rheumatism. In the *fibrous* form, the pain generally occurs sometime previous to the swelling, which is rather a puffiness from effusion into the cellular tissue, and exhibits pitting like general œdema. In the synovial variety, the swelling supervenes soon upon the pain and depends upon actual effusion into the cavity of the joint. The contour of the articulation is changed, and fluctuation frequently is detected in exposed joints. The redness is generally diffused equally over the part, while in the first species it often follows the course of the tendons. Let us refer to some of the characteristics of the two other diseases which I mentioned might occur at the joint, and are therefore to be borne in mind in forming our diagnosis in the present case. It is necessary only to allude to one or two of their distinctive features to assure ourselves that they do not exist in this boy. Acute synovitis occurs after puberty, from exposure to vicissitudes of temperature, and is a local disease affecting a single joint. Chronic or scrofulous synovitis is most common among young children, who present marks of strumous diathesis, is insidious in its incursion and tedious in its progress. We are led then, by exclusion, to attribute our patient's suffering to rheumatism which exhibits itself most strikingly in the right knee. In this opinion we are confirmed by the constitutional symptoms, indicating disturbance of the digestive functions, and by the number of articulations implicated. Now what *treatment* is to be pursued here? Before commencing the treatment of any case of disease, gentlemen, ask yourselves these questions. What do I wish to accomplish? What indications are to be met and followed? Don't prescribe for a name, nor label the boxes and bottles in your saddle bags, as I have known done—"good for rheumatism;" "good for fever and ague," &c. Such empiricism, in the profession, it is which has proved the "eccalabeon"—the egg-hatching process of not a little of the quackery that infests our land. But *revenons a nos moutons*. We have here general and local symptoms to combat, and our treatment must be pretty active. Let us correct the derangement of the digestive functions, and the local trouble will soon disappear. The patient should go home and go to bed. I would advise Pil Hydrarg. and Pulv. Doveri, aa. gr. v. at bed-time.

and in morning Tr. Sem. Colchici. m. xx. c. Magnes, Calc. ℥ss. in mixture. This will probably produce purging and relieve the rheumatism; I employ the magnesia here against the acid secretions, and you will find alkalies generally valuable in the treatment of rheumatism or gout. Locally, it is not a matter of much importance what you do, but it is well to envelope the part in oiled silk or flannel. Remember, the disense is a constitutional one, requiring canstitutional treatment and rest.

*Case 2d.—Scrofulous enlargement of lower extremity of femur in* a boy æt. 5 years. The right knee is considerably enlarged, the swelling being elastic and puffy, but not fluctuating, and preserving the form of the articulating extremity of the bone. The motion of the joint is somewhat impaired, and the boy walks with a limp. There is occasional pain in the part wh ch is not severe. The integuments over the part are discolored and the veins clearly perceptible. The complaint is attributed to a fall received sometime since. *Remarks by Dr. P.*—Although in the present case, the articulation is not primarily involved, it may become so secondarily, and I exhibit it as illustrating a point in pathology on which Nelaton has contributed much valuable information. This is one of the diseases popularly known as white swelling. It occurs in strumous patients, and you observe in this boy unmistakeable signs of scrofulous taint. The disease consists of a deposition of tuberculous matter, either encysted or infiltrated, in the cancellar structure of the bone. As the tendency of tubercle is to soften, ulceration may occur through the bone into the joint, or external to it, leading to repeated abscesses which penetrate the integuments, discharging their pus, containing a curd like substance. This may after a while become cheese-like in its consistence, resembling the matter found in scrofulous absorbent glands. The disease is very slow in its progress, and it is remarkable how little the patient sometimes suffers. even where the cartilages of the joint have ulcerated. This is very different, whon ulceration of these organs has proceeded from other causes. Sometimes, however, portions of bone become exfoliated, and lying in the cavity of the joint, give rise to excessive pain.

The *diagnosis* of this affection is easy in the first stage. When the joint becomes involved it may be mistaken for diseases arising from other causes, but the history of the case, the remarkable absence of pain in its commencement, the puffy elastic feel, from effusion into the cellular membrane, as distinguished from effusion into the joint itself; the tedious progress of the disease, and the scrofulous constitution, all assist us in arriving at a proper conclusion as to its nature. The *prognosis* is variable. Spontaneous recovery sometimes occurs even after the formation of abscesses and sinuses. In others the general health becomes seriously deranged, hectic ensues, and the patient sinks unless rescued by sacrificing the limb.

*Treatment.*—This disease, like the last we considered, is a constitutional one, and if forgetting this, you seek to cure your patient by local means alone, you will be disappointed in your efforts. The constitutional treatment should be such as is indicated by the scrofulous taint—generous diet, good air, the iodides of iron or potash, the wine of iron &c., &c. Locally, a variety of external applications are recommended, as blisters, escharotics of iodine, or ungiod. and hydrarg., &c., &c. I like verymuch free scarifications followed by poultices. Entire rest of the part is important. This can be effected by a splint. Sir B. Brodie recommends a pasteboard splint on each side of the limb, secured by a roller. Dr. Batchelder's plan, which I have frequently referred to, the compress sponge around the joint, covered with roller and kept wet, was used successfully in one case which came here. We will try in the present instance, and will probably be able to inform you of the result. The rationale of the operation of this plan, is absorption by continual pressure, from the swelling of the sponge.

We will remember, gentlemen, a case of scrofulous synovitis of the shoulder joint and another of the ankle, which presented themselves last Monday. I remarked at that time, that the first instance was a rather unusual one, this disease effecting other joints much oftener than the shoulder. Both patients were young children, each strongly scrofulous. The disease, in fact, almost always occurs within the first five years, advances slowly but steadily to absorption of the synovial membrane and cartilages and final destruction of the bone. In some cases the synovial membrane becomes fungeous. The various forms of this affection are so numerous at our weekly meetings, that you can scarcely require any present instruction as to its diagnosis and treatment. It is easily and early recognized by the pain produced on pressing the articular surfaces together. As to the treatment, if the hip be the seat of the disease, and you see it before suppuration has commenced, an issue back of the trochanter, kept open a long time, will do much good. If in the ankle, scarifications and poultices, followed by bandages, are useful. But let me again remind you of your general treatment and judicious regimen.

In acute synovitis, such as you saw in a woman at a recent meeting, you have an inflammatory affection requiring an antiphlogistic course—purgatives of pulv. purgans, and locally, leeching or cupping, until the inflammation is subdued. Then pressure by roller, or stimulating applications will be useful to procure absorption of effused matters.

*Case 3d.* In connection with the subject, I wish to draw your attention to the case of a man who complains of pain in his right hip, which commenced about two months since. There is nothing in the appearance of the patient indicating any serious disease. From the location of the pain, we might be led to suspect disease of the hip joint, but there is no increase of uneasiness when percussion or pressure is made on the trochanter, and the flexion of the limb is not impaired. When a joint is diseased, the patient is stiff in the morning and *thaws out*, as it were, as the day advances. This man feels pretty well in the morning, but becomes stiff and fatigued at night. Hence I conclude the trouble is not in the articulation. Let us inquire about the small of the back. The patient has pain there, which is worse when standing than when walking. This is always the case in mere debility of the part, while if inflammation were present, the uneasiness would be increased by motion. The man tells us he cannot rise easily and readily from a stooping posture, a fact often noticed among young men who masturbate. Our patient was married six months before the difficulty began, and it might be a question whether the latter had any connection with venereal indulgence. The pathology of this case is irritation from over taxation of the spinal nerves.

*Treatment.* Avoid any appreciable causes, use cold dash and friction to loins and hip; regulate bowels and diet.

